# Photoelastic analysis of stress distribution in single and three-unit implant-supported prostheses with different internal connections

**DOI:** 10.34172/joddd.025.42649

**Published:** 2025-09-30

**Authors:** Abbas Zahoui, Marcelo Coelho Goiato, Rodrigo Antonio de Medeiros, Daniela Micheline dos Santos, Marcio Campaner, Sandro Basso Bitencourt, Aldiéris Alves Pesqueira

**Affiliations:** ^1^Faculty of Dentistry, University Sagrado Coração – USC, Bauru, SP, Brazil; ^2^Department of Dental Materials and Prosthodontics, Araçatuba Dental School, UNESP, Araçatuba, São Paulo, Brazil; ^3^School of Health Sciences, University of Brasília, Brasília/DF, Brazil; ^4^Department of Rehabilitative & Reconstructive Dentistry, School of Dentistry, University of Louisville, Louisville, KY

**Keywords:** Dental implant, Biomechanics, Dental prosthesis

## Abstract

**Background.:**

The accurate fit between the dental implant and the prosthetic abutment is crucial for the stability of the entire prosthetic-implant system, providing a better distribution of the occlusal load to the surrounding bone. To improve stress distribution between the implant and bone, various types of internal connection implants have been introduced. However, few studies are available on the biomechanical behavior of these connections. This study investigated the stress distribution in screwed implant-supported prosthesis with different implant‒abutment connections using a photoelastic analysis.

**Methods.:**

Eight photoelastic models were fabricated in PL-2 resin and divided according to the different types of internal connections: Morse taper (MT), internal Morse hexagon (IMH), Morse taper hexagon (MTH), and frictional Morse taper (FMT) implants (3.75×11.5 mm), and the number of crowns (single and 3-unit pieces). Models were positioned in a circular polariscope, and 100-N axial and oblique (45º) loads were applied to the occlusal surface of the crowns using a universal testing machine. The stresses were photographically recorded and qualitatively analyzed using Adobe Photoshop software.

**Results.:**

Under axial loading, the number and distribution of high-intensity fringes did not differ among groups for both crown types (single and splinted 3-element). Low stress values were noted at the implant apex. The oblique loading increased the number of fringes for all groups. In conclusion, the internal connection tested in this study did not affect the number and distribution of stress.

**Conclusion.:**

The different types of internal connections provided better stability for the implant‒prosthesis set, which improved stress distribution when the prosthetic pillar was loaded, with the Morse cone friction system showing less stress. Oblique loading resulted in a higher stress concentration than axial loading.

## Introduction

 The use of osseointegrated dental implants provides new methods of prosthetic rehabilitation with high success rates, resulting in increased masticatory efficiency, satisfaction, and consequently, an improved quality of life.^1,2^ However, to achieve a successful implant rehabilitation treatment, in addition to biological factors related to implant‒tissue integration, the mechanical factors inherent to system design should also be considered, since a great part of implant failures occurs due to mechanical factors (90%).^1,3,4^

 Recent studies^1,2,5^ have shown that the type of connection between the abutment and implant is an essential parameter to evaluate the biomechanical behavior of implant-supported prostheses, since the accurate adaptation between the implant, abutment, and prosthetic components is responsible for the stability of the entire implant‒prosthetic system, providing better distribution of occlusal loads to the surrounding bone.^1,2,6-8^

 Therefore, it is crucial to understand each of these factors, their effect on stress distribution, and the interaction between them in order to optimize the masticatory load distribution through the prostheses, implants, and supporting bone.^6,8,9^

 To improve stress distribution between the implant and bone, various implant connection systems have been introduced. In implant-supported rehabilitations, two types of connections are primarily used: external (hexagons) and internal (hexagons, octagons, and conical/Morse taper [MT]), or a combination of both.^3,5,10^

 The internal connections stand out when compared to external ones, as they transfer lower stress to the implants when subjected to occlusal loads. Additionally, they present a lower incidence of screw loss, better absorption of external loads, and a more homogeneous stress distribution around the implants.^5,10^

 The remarkable step in the evolution of MT connections is the development of new connection designs. Currently, there are models of MT prosthetic abutment with different configurations: a solid body abutment, in which the screw and head consist of a single and indivisible part; and the transfixing screw prosthetic abutment, in which the conical head has an internal channel that allows the screw handling and implant torque to be carried out independently, presenting, in addition to the cone itself, stabilizing elements like threads locking and the presence of antirotacional hexagon.^3,4,8,11,12^

 Today, the significance of the biomechanical aspect in implant treatment has been emphasized, and safe measures have been sought to define the limits of stress transmission to dental implants.^1,2,4,5,8,10,13^

 Several studies^3,4,10,12,14^ have been conducted to introduce methods for assessing the behavior of bone tissue in the regions surrounding implants.

 Among these methods is the one adopted in this study: photoelastic analysis. In this technique, the stress location is observed in an experimental model through fringes of different colors. The amount of resulting deformation of a given force can be inferred by comparing the stresses observed with the area free of tension.^3-5,15,16^

 This study evaluated, through photoelastic analysis, the biomechanical behavior of single- and three-unit splinted implant-supported prostheses joined with different types of internal connections (MT, internal Morse hexagon [IMH], Morse taper hexagon [MTH], and frictional Morse taper [FMT]). This study hypothesizes that the various internal connection systems do not directly influence the stress distribution in both crowns (single and three-unit splinted).

## Methods

 A metallic matrix (40 × 45 × 10 mm) was obtained and poured with silicone (Sapeca Artesanato, Bauru, São Paulo, Brazil). The space created by the matrix was completed with type IV dental stone (Durone, Dentsply, Petrópolis, Rio de Janeiro, Brazil), which resulted in eight experimental blocks (44 × 22 × 10 mm) that were divided into eight groups according to the internal connection (CM, HIM, CMH, and CMF) and prosthesis (Table 1).

**Table 1:** Studied groups

**Models**	**Implant-abutment connection**	**Diameter (mm)**	**Prosthesis**
I	MT	11.5 × 3.75	Single
II	MT	11.5 × 3.75	3-unit piece
III	IMH	11.5 × 3.75	Single
IV	IMH	11.5 × 3.75	3-unit piece
V	MTH	11.5 × 3.75	Single
VI	MTH	11.5 × 3.75	3-unit piece
VII	FMT	11.5 × 3.75	Single
VIII	FMT	11.5 × 3.75	3-unit piece

Abbreviations: MT, Morse taper; IMH, internal Morse hexagon; MTH, Morse taper hexagon; FMT, frictional Morse taper (FMT).

 Models were perforated to receive implant replicas (DSP Biomedical, Campo Largo, Paraná, Brazil), using a parallelometer to standardize the insertion (in its long axis). The implant replica was screwed to the corresponding transfer (DSP Biomedical, Campo Largo, Paraná, Brazil) and inserted into the dental stone model until its platform reached the same level as the upper part of the block. The long axis of implant replicas was positioned perpendicular to the horizontal plane and fixed with autopolymerizing acrylic resin (Duralay, Duralay Reliance Dental, MFG Co Worth, IC, USA).^10,17^

 The blocks containing implant replicas were duplicated, and a new mold was obtained in which the implants of each group (DSP Biomedical, Campo Largo, Paraná, Brazil) were placed. Then, the mold was poured with photoelastic resin (PL-2, Vishay, Micro-Measurements Group Inc., Raleigh, NC, USA) according to the manufacturer’s recommendation. Each set was submitted to a 40 lbf/pol^2^ pressure to remove internal bubbles.^10,17^

 A total of eight blocks was obtained (blocks I, II, III, IV, V, VI, VII, and VIII).

 In blocks I, III, V, and VII, single-unit screwed crowns corresponding to the mandibular second premolar were fabricated. In contrast, for blocks II, IV, VI, and VIII, 3-unit screwed crowns corresponding to mandibular second premolar, first molar, and second molar were installed; all of them were fabricated with standardized dimensions, in nickel-chromium alloy (Fit Cast –SB Plus, Talladium do Brasil, Curitiba, PR, Brazil). A 20-N torque was applied for screwing crowns to the implants, according to the manufacturer’s instructions.^10^

 The set was placed in a circular polariscope, and an initial photograph was taken without load application to verify the absence of stresses on the photoelastic blocks. Thereafter, 100-N axial and oblique (45º) loads were applied individually, on the occlusal surface of all crowns, for 10 seconds, through a universal testing machine (EMIC-DL 3000, São José dos Pinhais, Paraná, Brazil). A 45º tilted apparatus was used for oblique load application. Data were photographically recorded (Nikon D80, Nikon Corp, Japan) and analyzed in an image software (Adobe Photoshop CS3, San Jose, California, USA).^10,17^

 To verify the stress direction and intensity, photographic records of all blocks were qualitatively analyzed. The images were interpreted as follows: the higher the fringe order (N) and number, the greater the stress intensity. Moreover, the closer the fringes were to each other, the higher the stress concentration. The measurement was based on the high-intensity fringe number count (green‒pink transition = 696 kPa).^5,10,15^

 The obtained images were classified according to the number of fringes and the concentration of tension of each sample. For the number of fringe analyses, it was verified that fringes of moderate (green-red) and high tension (green-pink) were present. The same operator evaluated all the images.

###  Statistical Analysis

 Due to the qualitative nature of photoelastic analysis, no statistical test was performed. The results were interpreted based on the visual assessment of stress fringes, as commonly reported in similar studies.^3,4,10,13^

## Results

 Overall, all the groups exhibited similar formation of fringes (Table 2). A higher concentration of tension was observed in the apical region of the implants, particularly in the distal implant, where the formation of fringes was more pronounced (Figures 1-8).

**Table 2:** Corresponding voltage values (transition between green/pink = 696 kPa) to the number of fringes, according to each evaluation group and region, according to the point (element) of load application

**Blocks**	**Axial loading fringe number **			**Oblique loading fringe number **		
	**Element**			**Element**		
	**34**	**35**	**36**	**34**	**35**	**36**
I		696- (1)			4.872-(7)	
II	1392- (2)	0	1392-(2)	1392- (2)	0	4176–(6)
III		1392- (2)			4176 -(6)	
IV	1392- (2)	0	1392- (2)	2784- (4)	0	4.176-(6)
V		696- (1)			4.176-(6)	
VI	1392- (2)	0	1392- (2)	4.872-(7)	0)	4176 -(6)
VII		696 –(1)			1392 (2)	
VIII	696- (1)	0	1392- (2)	1392- (2)	0	2088-(3)

**Figure 1:**
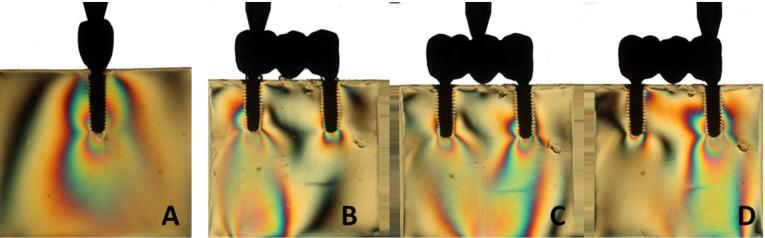
(A-D) Axial load for blocks I and II

**Figure 2:**
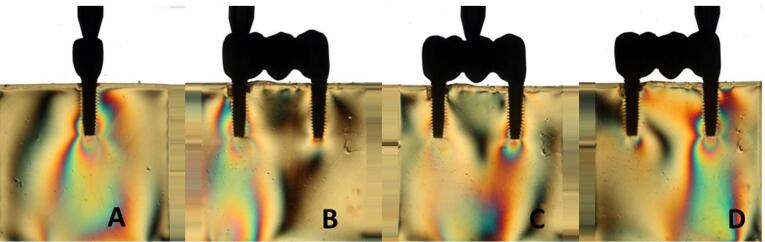
(A‒D) Axial load for blocks III and IV

**Figure 3:**
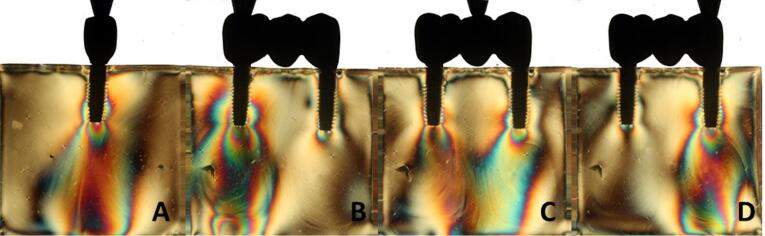
(A‒D) Axial load for blocks V and VI

**Figure 4:**
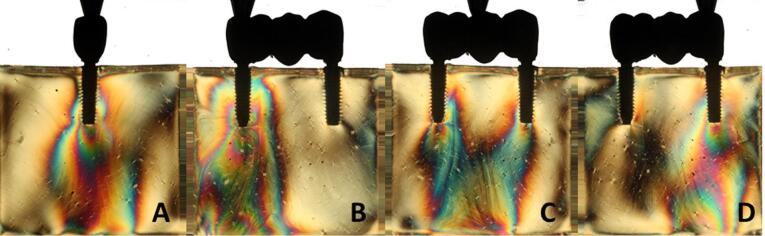
(A‒D) Axial load for blocks VII and VIII

**Figure 5:**
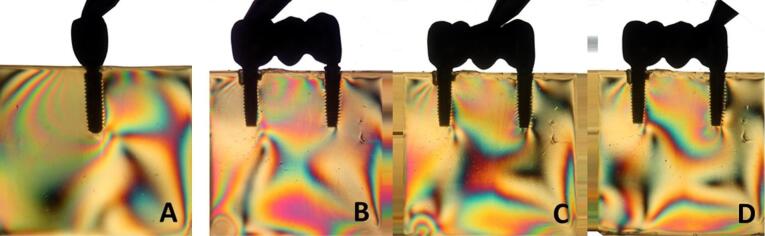
(A‒D) Oblique load for blocks I and II

**Figure 6:**
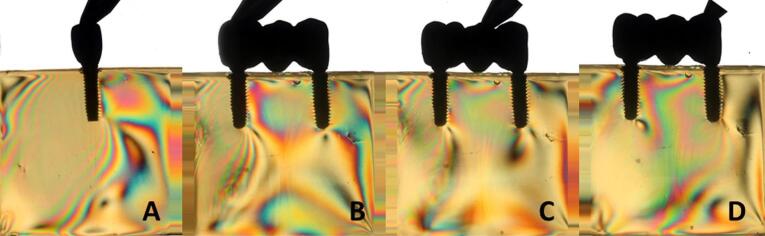
(A‒D) Oblique load for blocks III and IV

**Figure 7:**
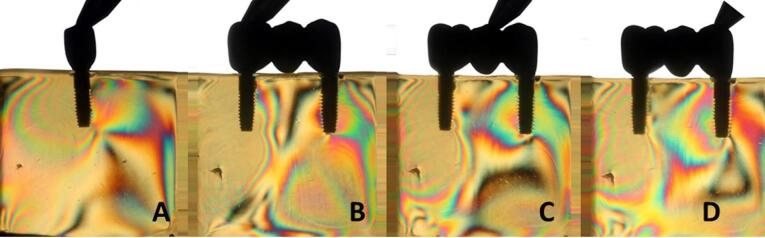
(A‒D) Oblique load for blocks V and VI

**Figure 8:**
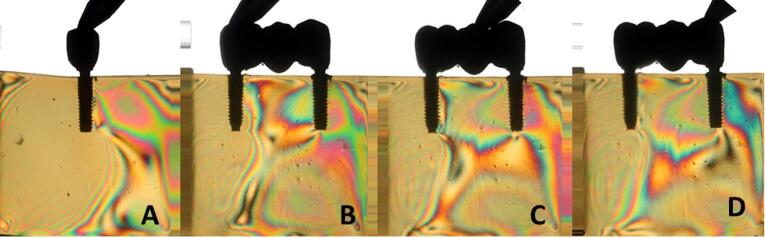
(A-D) Oblique load for blocks VII and VIII

 The results (Table 2) for single-unit crowns showed that groups I (Figure 1A), V, and VII (Figure 3A) exhibited the lowest number of fringes (n = 1). Group III (Figure 4A) presented results (n = 2). In the three-unit splinted crowns, all the groups showed a similar number of fringes (Figures 1‒4). Oblique loading increased the number of fringes in all groups (Figures 5-8), with the single-unit crown groups (I, III, V, and VII) (Figures 5A, 6A, 7A, and 8A) presenting higher stress values than the three-unit splinted crown groups (Figures 5B-D, 6B-D, 7B-D, and 8B-D). In both axial (Figures 1-4) and oblique (Figures 5‒8) loads, all blocks presented similar stress distribution patterns regardless of the type of connection and prosthesis, except for group VIII (Figure 8), which demonstrated lower stress values. It was observed that the stresses were concentrated at the apex of the implant (Figures 1‒8). However, for the oblique load in the groups with three-unit splinted crowns (II, IV, VI, and VIII), the stresses were also concentrated at the implant neck (Figures 5B-D, Figure 6B-D, Figure 7B-D, and Figure 8B-D).

## Discussion

 The hypotheses that different internal connection systems directly influence the stress distribution in both crowns (single and three-unit) and that the FMT system presents less stress were not accepted in this study, since there was no difference in the number (Table 2) and distribution (Figures 1-8) of high-intensity fringes between the groups tested in both crowns (single and three-unit).

 Photoelastic models are an alternative for understanding biomechanics in health sciences; they visually provide qualitative information, particularly about the origin and direction of stress.^10,18^

 The Morse cone frictional system presents less stress and offers a better adaptation between the implant and the abutment, with internal walls that are almost parallel or inclined at 8º or 11º, forming a tight and strong union as if it were a “cold welding” or a one-piece fit.^11^

 Several studies^7,19^ have shown that potential misfits between implants and abutments can cause mechanical problems as occlusal forces may exceed physiological bone limits, leading to failures in rehabilitation, such as preloading loss, fracture of prosthetic components and screws, or even loss of osseointegration. Hekimoglu et al^20^ highlighted that excessive loads on the bone‒implant interface are one of the key factors responsible for marginal bone loss, motivating studies on microdeformations, which allow the definition of parameters that verify harmful levels to this interface.

 In our study, there was no difference in the number of high-intensity fringes between the tested groups (Table 2 and Figures 1‒4) since all the groups exhibited low stress values. These results are probably related to the biomechanical superiority of internal connection, which corroborates with other studies^2,3,5,10^ that reported the advantages of using MT connection system, such as the absence of microcracks at abutment‒implant connection because of an accurate fit between the conical surfaces, what also decreases the risk of gap formation and prevents bacterial contamination; better stress transmission from the abutment to the implant, since it causes a better distribution of masticatory loads on the internal walls of the implant, what makes the tension gradient on cervical region to be transmitted to the bone more adequately, protecting the prosthetic abutment retaining screw and avoiding its loosening; better stability of the abutment‒implant connection and lower bone loss; the cone angle is produced accurately, causing great friction retention, hampering the removal of the abutment from the respective implant.^16,17^

 The superiority of the MT system in stress distribution was also reported by Goiato et al^3^ in 2014, who used photoelastic analysis to evaluate the biomechanical behavior of implant-supported screwed prostheses with different connection systems (external hexagon (HE) and MT), with single crowns or three-unit splinted crowns. The results showed that a single crown with the MT system presented a lower number of fringes than with the HE system in both loads (axial and oblique). However, in the three-unit splinted crowns (MT), fringe values were lower. All groups showed an increased number of fringes when the oblique load was applied. The authors concluded that the type of implant connection system directly influenced stress distribution in both single and three-unit splinted crowns, and the best system for a single prosthesis was MT.

 Other studies^5,7,12^ have also shown that such internal connections distribute stresses more uniformly, once it is possible to create a deeper connection and increased contact between the abutment and the internal walls of the implant, providing the abutment a better fit and reducing the possibility of micromovements. They also have advantages such as an easy connection with the abutment, greater stability, and anti-rotation, making them indicated for single restorations. They offer greater resistance to lateral loading due to a lower rotation center and better stress distribution.

 Furthermore, in the MT systems, the conical juxtaposition in the implant‒abutment interface is located at the center of the implant platform, distant from the alveolar bone, differently from the external hexagonal system, in which the abutment is positioned all over the implant platform and the interface with the abutment is adjacent to bone tissue. Still, the conical systems present a large contact area and frictional resistance at the implant‒abutment interface, providing a safe connection.^3,4,11,14^ They exhibit excellent mechanical stability, evidenced by in vitro studies^11,12,21^ and longitudinal clinical follow-ups, in addition to hindering the abutment removal and providing them with antirotational properties, without needing geometrical designs, such as a hexagonal platform used in the hexagonal system.^12^

 Currently, the success or failure of treatment with dental implants is primarily determined by the way stresses are transmitted and absorbed by the surrounding bone tissue. There is a large number of publications about the effects of implant position and angulation, the magnitude and direction of the load, implant design (shape, length, and diameter), surface characteristics, the prosthesis type, the quantity and quality of surrounding bone tissue, and the type of prosthetic connection, on the distribution of stresses in implant-supported prostheses.^6,9^

 In the application of oblique load, all the blocks presented an increased number of fringes (Table 2 and Figures 5‒8). It was also observed that stresses were concentrated at the implant apex (Figures 1‒4). In groups with three-unit splinted crowns (II, IV, VI, and VIII), stresses were also concentrated in the region of the implant neck (Figures 5B-D, 6B-D, 7B-D, and 8B-D). It is known that oblique loads, especially, generate stresses in the implant cervical region, which can facilitate the process of bone resorption and component fracture, as demonstrated by Naveau et al,^22^ who reported that cervical bone loss is related to stress concentration at this level.

 Two types of loads that act on implants must be specially considered: axial and oblique. The axial load is more advantageous as it distributes stresses more uniformly across the implant’s long axis, in contrast to the oblique load, which generates higher stresses over the implant and bone tissue.^23^

 The biomechanical influence of internal connection systems has not been totally clarified. Different types of internal connections are available in the market, although there is no consensus in the literature about the best alternative. Therefore, further studies are necessary to assess the influence of different internal connections on the stress distribution in the prosthesis/abutment/implant/bone complex, searching for a better predictability in stress distribution and to provide the clinician with answers about which connections have better behavior on stress distribution in bone tissue and which system would present lower potential for bone loss.

 The limitations of this study are primarily due to its in vitro nature, which affords greater control during testing and eliminates the adversities encountered in a clinical study. Future studies using other methodologies, such as finite element analysis and strain gauge analysis, are recommended for a more accurate assessment of stress distribution patterns in bone to complement the results, as well as to study how different implant shapes and sizes and the use of an oblique load may influence other types of implant platforms.

## Recommendations

 All implant connections in this in vitro study demonstrated acceptable photoelastic analysis of stress distribution; however, in this in vitro study, we recommend the use of MT connections for single-abutment prostheses after both loads (axial and oblique) and three-unit implants both loads (axial and oblique) the FMT connection, as these showed low stress values, resulting in less stress dissipation around the implants.

## Conclusion

 It was concluded that the different internal connection systems evaluated provided greater stability to the entire prosthetic/implant system, with an acceptable distribution of stresses when the abutment was subjected to stress, with the Morse cone friction system showing less stress. Oblique loading produced a greater concentration and intensity of stresses than axial loading.

## Competing Interests

 The authors declare that they have no conflicts of interest related to this study.

## Data Availability Statement

 The data underlying this article will be made available by the authors upon reasonable request.

## Ethical Approval

 Not applicable since this was performed in vitro.
